# Syringaresinol Inhibits UVA-Induced MMP-1 Expression by Suppression of MAPK/AP-1 Signaling in HaCaT Keratinocytes and Human Dermal Fibroblasts

**DOI:** 10.3390/ijms21113981

**Published:** 2020-06-01

**Authors:** Jung Hwan Oh, Yung Hyup Joo, Fatih Karadeniz, Jaeyoung Ko, Chang-Suk Kong

**Affiliations:** 1 Marine Biotechnology Center for Pharmaceuticals and Foods, College of Medical and Life Sciences, Silla University, Busan 46958, Korea; wjdghks0171@naver.com (J.H.O.); karadenizf@outlook.com (F.K.); 2 AMOREPACIFIC Research and Development Center, Yongin 17074, Korea; yunghyupjoo@hanmail.net; 3 Department of Food and Nutrition, College of Medical and Life Sciences, Silla University, Busan 46958, Korea

**Keywords:** HaCaT keratinocytes, human dermal fibroblasts, MMP-1, photoaging, UVA

## Abstract

Ultraviolet (UV) irradiation induces detrimental changes in human skin which result in photoaging. UV-induced intracellular changes cause degradation of extracellular matrix (ECM). UV-stimulated cleavage of collagen in ECM occurs via matrix metalloproteinases (MMPs). (±)-syringaresinol (SYR), a phytochemical which belongs to the lignan group of polyphenols, was investigated for its ability to reverse the UVA-induced changes in human HaCaT keratinocytes and dermal fibroblasts (HDFs) in vitro. Effect of SYR on UVA-induced changes was investigated by production and activation of MMPs and its transcriptional upstream effectors; mitogen-activated protein kinases (MAPKs) and pro-inflammatory mediators. Levels of expression were determined using ELISA, RT-PCR and immunoblotting. UVA irradiation stimulated the production of MMP-1 and inhibited collagen production. SYR treatment suppressed MMP-1 and enhanced collagen production in UVA-irradiated HaCaT keratinocytes and HDFs. SYR repressed the UV-induced phosphorylation of p38, ERK and JNK MAPKs in HaCaT keratinocytes while only suppressing JNK phosphorylation in HDFs. In addition, SYR was able to inhibit UVA-induced production of inflammatory cytokines; TNF-α, COX-2, IL-1β and IL-6. Moreover, SYR suppressed the activator protein-1 (AP-1), a heterodimer of phosphorylated transcription factors c-Jun and c-Fos. SYR-treatment decreased nuclear levels of activated c-Fos and c-Jun as a mechanism to inhibit UVA-induced transcriptional activities leading to MMP-1 production. In conclusion, current results demonstrated that SYR could inhibit UVA-induced upregulation of MMP-1 by suppressing MAPK/AP-1 signaling in HaCaT keratinocytes and HDFs. Therefore, SYR was suggested as a potential compound with antiphotoaging properties against UVA-induced skin aging.

## 1. Introduction

Ultraviolet (UV) radiation exposure leads to several skin problems, such as wrinkles, loss of elasticity, sagging, and deformation [1,2]. Chronic exposure to UV-induced changes in skin is considered to be the root cause of extrinsic skin aging, therefore referred to as photoaging [3]. Photoaging skin was observed to exhibit UV-induced deterioration, mainly in dermal connective tissues: epidermis and dermis layers. Ultraviolet A (UVA) is one of the light rays that are emitted by sun together with UVB and UVC. UVB has the most detrimental and broad harmful effects and affects mainly the top layer of skin, causing sunburn, redness and cancer. UVA irradiation, on the other hand, can penetrate deep layers of skin and is the main cause of premature skin aging and wrinkling [3,4]. Further, it has been shown that exposure to UV radiation also affect the neuroendocrine system through skin exposure [5]. This interaction between UVR and the neuroendocrine system shows itself in different ways including opioidergic effects, immunosuppression, and brain stimulation, all of which open ways for UVR to be used in a therapeutic approach. However, it was also shown that premature aging of the skin could also be enhanced by UVR-mediated stimulation of neuroendocrine system by activating internal aging factors in addition to its extrinsic aging stimulatory effects [6]. Research has targeted the molecular changes after UVA exposure in skin. It was shown that, after a short time of exposure, UV radiation activated growth factors, increased oxidative stress and induced pro-inflammatory response in different types of skin cells [7,8]. Several of these outcomes are known to enhance the activation of mitogen-activated protein kinase (MAPK) cascade, which is followed by an increase in transcriptional activity of the activator protein-1 (AP-1) complex formed by heterodimerization of c-Fos and c-Jun proteins [9]. Activity of the AP-1 complex is dependent on the activation of c-Fos and c-Jun proteins by MAPK cascade and responsible for the upregulation of several extracellular-matrix-degrading (ECM) enzymes, such as matrix metalloproteinase-1 (MMP-1), MMP-3 and MMP-9 [10]. Therefore, UV-induced degradation of ECM, especially its collagen fragments, is one of the main causes of photoaged skin.

**Figure 1:**
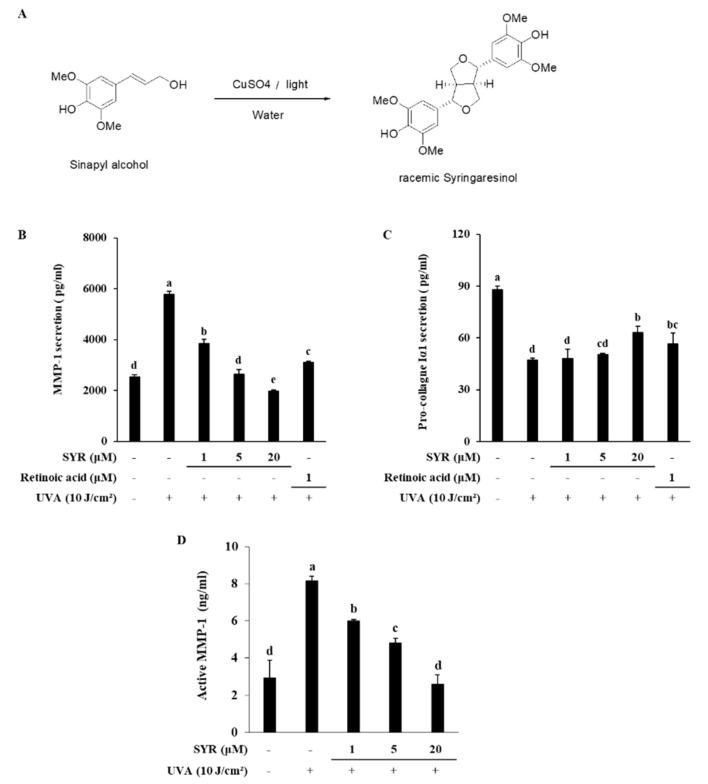
Synthesis of (±)-syringaresinol (SYR) (**A**) and its effect on UVA-induced release of MMP-1 and procollagen Iα1 in HaCaT keratinocytes. Cells were treated with given concentrations of SYR for 24 h after UVA irradiation (10 J/cm^2^), and levels of MMP-1 (**B**) and procollagen Iα1 (**C**) release along with MMP-1 activation (**D**) were measured. Retinoic acid was used as a positive control. Values are means ± SD of three independent experiments run in triplicate. Different letters (a–e) indicate statistically significant difference at *p* < 0.05 level by Duncan’s multiple range test.

Studies during the past decades showed an increasing interest in naturally occurring compounds, especially phytochemicals, given their broad-spectrum bioactivities, high biodegradability, low cost and low toxicity [11]. Lignans belong to a group of such phytochemicals found in various plants and include numerous biologically active compounds with pharmaceutical value [12]. Syringaresinol belongs to the lignan group of phytochemicals and exists as (R, R)-(+)-form and (S, S)-(-)-form. It can be obtained by extracting various parts of plants, such as cortex, Amelia seed and flax seed. This substance has been reported to show various physiological activities, such as antibacterial, antifungal activity [13] and anti-inflammatory [14] activity, and recently, research results related to skin, such as whitening, anti-aging, improvement of immune disease, and improvement of metabolic disorders [15] are known. Therefore, the current study was carried out to investigate the effects of (±)-syringaresinol (SYR) (Figure 1A) in UVA-irradiated human keratinocyte and dermal fibroblast in order to provide insights regarding its antiphotoaging properties.

## 2. Results

### 2.1. SYR Reversed UVA-Induced Changes of MMP-1 and Procollagen Iα1 Production in HaCaT Keratinocytes

**Figure 2:**
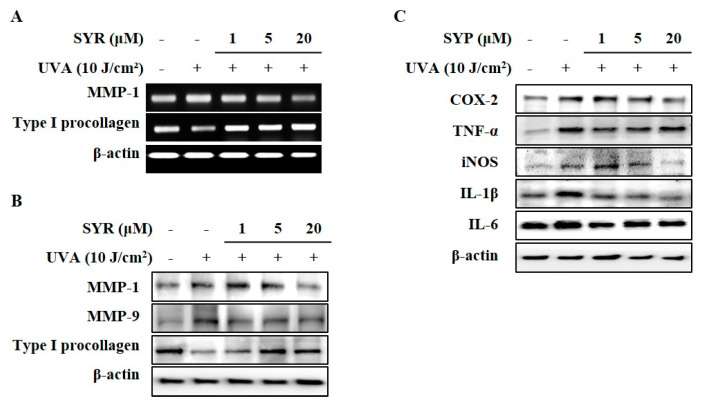
Effect of SYR on UVA-induced mRNA and protein expressions of MMPs, type I procollagen, and pro-inflammatory mediators in HaCaT keratinocytes. Cells were exposed to UVA radiation (10 J/cm^2^) and treated with given concentrations of SYR for 24 h. (**A**) mRNA expressions of MMP-1 and type I procollagen were analyzed with RT-PCR. (**B**) Protein levels of MMP-1, -9 and type I procollagen (**C**) and pro-inflammatory mediators (COX-2, TNF-α, iNOS, IL-1β and IL-6) were determined by Western blotting. β-Actin was used as an internal control for RT-PCR and Western blotting. Retinoic acid was used as a positive control.

To examine the effects of UVA irradiation on the production of MMP-1 and procollagen Iα1 in keratinocytes and whether SYR could counter these effects, HaCaT keratinocytes were irradiated with UVA and treated with different concentrations (1, 5 and 20 μM) of SYR. UVA exposure significantly induced MMP-1 production in keratinocytes (Figure 1B). On the other hand, release of procollagen Iα1 was inhibited by UVA exposure (Figure 1C). Treating keratinocytes with SYR significantly inhibited MMP-1 production in a dose-dependent manner while increasing the release of procollagen Iα1 from keratinocytes compared to UVA-irradiated non-treated cells. SYR was also shown to decrease the activation of MMP-1 in cell culture medium (Figure 1D). A UVA-induced increase in active MMP-1 levels (8.16 ng/mL) was significantly decreased (2.56 ng/mL) by 20 μM SYR treatment. Similar effects were observed in the expression of MMP-1 and type 1 procollagen in both mRNA and protein level. SYR treatment was able to reverse the effects of UVA exposure on mRNA expression (Figure 2A) and protein levels (Figure 2B) of both MMP-1 and type 1 procollagen. Dose-dependently, MMP-1 expression was inhibited by SYR after notable UVA-induced upregulation (Figure S1). Likewise, UVA-induced suppression of type I procollagen expression was reverted by SYR. In addition, to determine the effects of SYR on the UVA-induced expression of other MMPs with the same transcriptional regulation (AP-1), MMP-9 protein levels were analyzed (Figure 2B). Expectedly, UVA irradiation upregulated MMP-9 levels, which were decreased by the presence of SYR, suggesting that the effect of SYR on UVA-induced MMP production was not MMP-type specific.

### 2.2. UVA-Induced Inflammation in HaCaT Keratinocytes is Ameliorated by SYR Treatment

As TNF-α mediated inflammatory cytokines can also stimulate MMP-1 production and collagen degradation [16]; effects of SYR treatment against UVA-induced inflammatory response in keratinocytes were analyzed via protein expression of pro-inflammatory mediators and cytokines (TNF-α, COX-2, iNOS, IL-1β and IL-6). Keratinocytes exhibited increased levels of TNF-α, COX-2, iNOS, IL-1β and IL-6 following UVA exposure (10 J/cm^2^) (Figure 2C). UVA-induced stimulation of inflammatory cytokine production was inhibited by SYR in a dose-dependent manner except for IL-6.

### 2.3. SYR Suppresses the UVA-Induced Activation of MAPK/AP-1 Signaling in Keratinocytes

**Figure 3:**
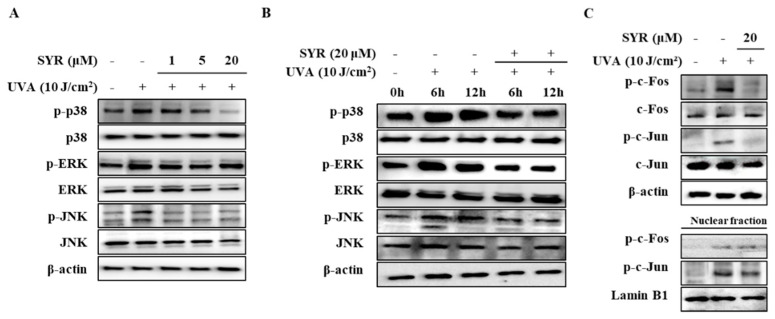
Effect of SYR on the total and phosphorylated (p-) protein levels of MAPK/AP-1 signaling effectors in UVA-irradiated HaCaT keratinocytes. Total cell lysates and nuclear extracts of the cells were used to determine the protein levels of phosphorylated (p-) and inactive forms of (**A**,**B**) MAPKs (p38, ERK and JNK), (**C**) c-Fos and c-Jun proteins by Western blotting. Cells were exposed to UVA radiation (10 J/cm^2^) and treated with or without given concentrations of SYR for 24 h (**A**,**C**), 6 h and 12 (**B**). β-Actin was used as an internal control for Western blotting. Lamin B1 was used as an internal control for nuclear fraction Western blotting.

As activation of p38, ERK and JNK MAPKs are required for upregulating MMP-1 expression [17] via UVA-induced ROS production, the effects of SYR on the phosphorylation levels of MAPKs were investigated (Figure S2). HaCaT keratinocytes were irradiated by UVA (10 J/cm^2^) followed by SYR treatment. Levels of phosphorylated (p-) p38, p-ERK and p-JNK were significantly increased by UVA exposure (Figure 3A). Treatment with SYR inhibited the p- levels of all three MAPKs without affecting their total protein levels, indicating that SYR downregulated the UVA-stimulated phosphorylation of MAPKs, not their expression. Time-dependent activation of MAPK was also investigated after 6 and 12 h of UVA irradiation and treatment. SYR treatment (20 μM) showed decreased phosphorylation of p38, ERK and JNK during the early stages of UVA irradiation, further suggesting the effect of SYR on MAPK phosphorylation (Figure 3B).

UVA-induced AP-1 activation and nuclear translocation were shown to result in increased MMP-1 expression due to AP-1 binding site presence on the MMP-1 promoter region [18]. Given that AP-1 is formed by Jun and Fos proteins and its activation is regulated by MAPKs, the effects of SYR on UVA-mediated c-Jun and c-Fos phosphorylation were determined. UVA irradiation (10 J/cm^2^) resulted in elevated phosphorylation of c-Jun and c-Fos (Figure 3C) both in the cytoplasm and nucleus of keratinocytes. Treating UVA-irradiated HaCaT keratinocytes with 20 μM SYR inhibited UVA-induced phosphorylation of c-Fos and c-Jun, as well as suppressing their nuclear translocation, as shown by inhibited levels of p-c-Fos and p-c-Jun in the nuclear fraction of UVA-irradiated keratinocytes. Coupled with suppressed MAPK activation, inhibition of c-Fos and c-Jun phosphorylation suggested that SYR inhibited MMP-1 expression and, thus, MMP-1 production via downregulated AP-1 transcriptional activity.

### 2.4. SYR Inhibits the UVA-Induced MMP-1 Production in Human Dermal Fibroblasts (HDFs) by Inhibiting MAPK-Mediated Pro-Inflammatory Response and AP-1 Activation

**Figure 4:**
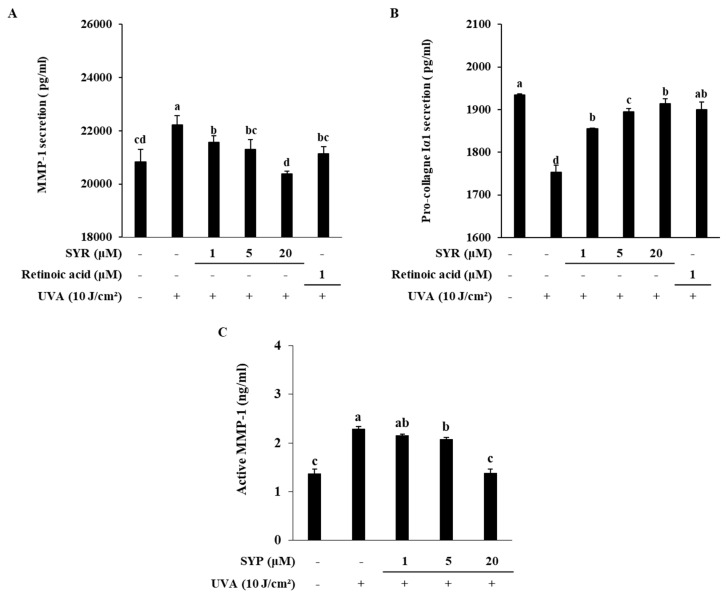
Effect of SYR on UVA-induced release of MMP-1 (**A**) and procollagen Iα1 (**B**) along with MMP-1 activation (**C**) in human dermal fibroblasts (HDFs). Cells were treated with given concentrations of SYR for 24 h after UVA irradiation (10 J/cm^2^), and the levels of MMP-1 and procollagen Iα1 release were measured by ELISA using cell culture medium. Retinoic acid was used as a positive control. Values are means ± SD of three independent experiment run in triplicate. Different letters (a–d) indicate statistically significant differences at the *p* < 0.05 level by Duncan’s multiple range test.

**Figure 5:**
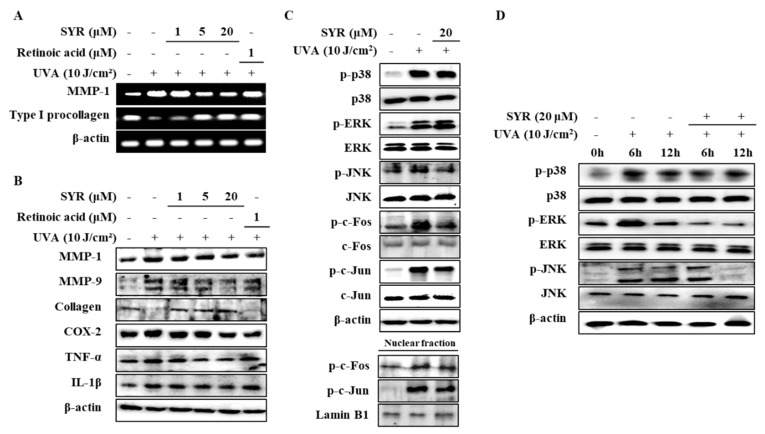
Effect of SYR on the expression of MMPs, type I procollagen, pro-inflammatory cytokines and MAPK/AP-1 phosphorylation in UVA-irradiated HDFs. (**A**) mRNA expressions of MMP-1 and type I procollagen were determined by RT-PCR. (**B**) Protein levels of MMP-1, MMP-9, collagen, COX-2, TNF-α, and IL-1β were analyzed with Western blotting using total cell lysates. (**C**,**D**) Phosphorylated (p-) and total levels of p38, ERK, JNK, c-Fos and c-Jun were analyzed with Western blotting using total cell lysates and nuclear extracts. Cells were exposed to UVA radiation (10 J/cm^2^) and treated with given concentrations of SYR. After 24 h (**A**–**C**) or 6 and 12 h (**D**) incubation cells were harvested for RT-PCR and Western blotting. Retinoic acid was used as a positive control. β-Actin was used as an internal control for RT-PCR and Western blotting. Lamin B1 was used as an internal control for nuclear fraction Western blots.

To investigate whether attenuation of UVA-induced MMP-1 production and collagen degradation by SYR was cell-type-specific, HDFs were treated with SYR following UVA irradiation (10 J/cm^2^). Similar to HaCaT keratinocytes, HDFs showed increased MMP-1 production coupled with decreased procollagen Iα1 release (Figure 4A,B). Treating UVA-irradiated HDFs with SYR resulted in reverted production levels for both MMP-1 and procollagen. SYR-treated HDFs released less MMP-1 and more procollagen compared to that of the UVA-irradiated-only group. Similar to its effect on HaCaT keratinocytes, SYR suppressed UVA-induced MMP activation (Figure 4C). At the concentration of 20 μM, SYR treatment decreased the active MMP-1 levels from 2.28 ng/mL (untreated UVA-irradiated group) to 1.37 ng/mL. Parallel results were obtained from investigating mRNA and protein expression of MMP-1 and type I procollagen. SYR ameliorated the change in MMP-1 and type I procollagen mRNA expression (Figure 5A) in UVA-irradiated HDFs. Likewise, MMP-1 and collagen protein levels were reversed to non-irradiated non-treated HDF group levels by SYR compared to the UVA-irradiated-only group (Figure 5B). In addition, MMP-9 protein levels were also slightly inhibited by SYR in a dose-dependent manner. The protein levels of pro-inflammatory mediators in UVA-irradiated HDFs were also analyzed with or without SYR treatment to determine whether SYR could inhibit UVA-induced inflammation in HDFs as well. Treating HDFs with SYR following UVA irradiation inhibited the protein levels of TNF-α, COX-2, iNOS and IL-1β in HDFs (Figure 5B). As in keratinocytes, dermal fibroblasts were shown to exhibit overactivated p38, ERK and JNK MAPKs following UV exposure as a mechanism to express AP-1-regulated MMP-1. Presence of SYR (20 μM) inhibited the phosphorylated levels of JNK but did not suppress the p38 and ERK phosphorylation levels (Figure 5C). Analysis of MAPK activation at the early stages of UVA irradiation showed that SYR treatment was able to decrease the phosphorylation of ERK at the 6 and 12 h periods after UVA irradiation (Figure 5D). However, treatment with SYR enhanced the activation of ERK in HDFs at the 24 h period. Nevertheless, SYR was able to hinder the phosphorylation of c-Fos and c-Jun and nuclear translocation of activated c-Jun but not c-Fos (Figure 5C) in nuclear fractions of UVA-irradiated HDFs. Results indicated that SYR reversed the UVA-induced production of MMP-1 in HDFs via a similar mechanism to that of HaCaT keratinocytes: repression of inflammation and MAPK-activated AP-1 transcriptional activity (Figure S3).

## 3. Discussion

UV-irradiation-induced skin aging concerns the deterioration of the balance between MMP production and ECM formation in favor of MMPs, which results in increasing degradation of ECM by various cell types including dermal fibroblasts and keratinocytes [2]. UVA is known to induce the production and release of MMP-1 and MMP-9 in epidermal and dermal layers of skin, resulting in degradation of collagen. Considering the importance of collagen in skin function and elasticity, UVA-induced collagen degradation can be regarded as the main path for the progression of photoaging. In present study, it was shown that SYR treatment dose-dependently inhibited the UVA-induced production of MMP-1 both in keratinocytes and HDFs. Results also showed that UVA irradiation inhibited the procollagen release from cells, which might be the reason behind chronic collagen deficiency in photodamaged skin, along with increased collagen degradation by MMPs. Present results demonstrated that UVA-induced decreases in procollagen release from HaCaT keratinocytes and HDFs were also attenuated by SYR. Immortalized HaCaT keratinocytes were chosen for the current study in order to minimize the problems and variations that come with primary keratinocyte cultures due to donor to donor differences and different characteristics for cells with different passage numbers. As current study is the first report of SYR UVA protective effects on keratinocytes, a standardized and long-lived HaCaT keratinocytes were used to obtain preliminary results. However, due to possible limitations and transcriptional differences between immortalized HaCaT keratinocytes and primary keratinocyte cultures, further studies using primary cultures and in vivo models are needed to fully evaluate the potential of SYR.

The effect of SYR on the production of MMP-1 and procollagen was also observed in expressional level. SYR-treated keratinocytes and HDFs showed decreased MMP-1 and increased collagen mRNA and protein levels compared to UVA-irradiated-only cells. SYR treatment was also observed to result in significantly decreased amounts of active MMP-1, indicating suppressed MMP-1 activation. 

UVA-induced expression of MMP-1 is mainly governed by MAPK-regulated activation of AP-1 and NF-κB, the former as a result of UVA-induced inflammatory response in skin [19,20]. On the other hand, AP-1 transcriptional activity is regulated by p38, ERK and JNK-mediated phosphorylation of c-Fos and c-Jun, which form the AP-1 complex upon phosphorylation. Therefore, the MAPK signaling cascade plays pivotal roles in UVA-induced MMP expression. This study showed that treating keratinocytes and HDFs with SYR inhibited the phosphorylation of p38, JNK and ERK MAPKs, with the exception of ERK in HDFs. Inhibition of MAPK activation by SYR was also shown to occur, along with inhibition of production of inflammatory cytokines such as TNF-α, COX-2, IL-1β and IL-6, all of which are part of the UVA-induced inflammatory response in skin, leading to production of MMPs. SYR treatment also significantly inhibited the phosphorylation of c-Jun and c-Fos and the levels of active c-Fos and c-Jun in nuclear fractions in UVA-irradiated keratinocytes and HDFs. Suppression of c-Fos and c-Jun activation indicated that SYR might inhibit MMP-1 production via blocking MAPK-cascade-regulated AP-1 transcriptional activity. Considering SYR inhibited the inflammatory cytokine production along MMP-1 expression, it was suggested that the effects of SYR targeted upstream activators of AP-1 and inflammation response, suggestive of phosphorylation of p38 and JNK. The stimulatory effects of SYR on collagen production in HDFs might be attributed to enhanced ERK activation, as some studies reported that dermal fibroblast collagen production is dependent on ERK1/2 signaling and negatively regulated by p38 [21,22]. However, SYR treatment was shown to suppress ERK phosphorylation at the early stages of treatment following UVA irradiation. Time-dependent opposite effects of SYR on ERK phosphorylation also suggest that the SYR-mediated stimulation of collagen synthesis via ERK activation occurred after downregulation of MMP-1 expression. Results indicated that SYR repressed MAPK activation independent of the stimuli, as both inflammatory and AP-1-mediated transcriptional activities were suppressed. SYR also showed its MAPK inhibitory effects in both keratinocytes and HDFs, demonstrating that inhibition of MMP-1 production via MAPK suppression was not cell-type-specific. On the other hand, SYR stimulated ERK phosphorylation in collagen-producing HDFs but not in keratinocytes, suggesting that SYR-mediated amelioration of UVA-induced changes in collagen production might be linked with ERK activation.

Lignans are important phytochemicals with various health benefits, including skin protection and skin whitening [23,24]. Syringaresinol has already been reported to be an important lead molecule with tyrosinase inhibitory [15] and antiaging [25] activities along with age-related skin atrophy reversion [26]. These reports showed that compositions containing syringaresinol and its derivatives as main ingredients possessed skin-improving abilities with antioxidant properties. In this context, the current study provided insights into the anti-photoaging properties of syringaresinol against UVA-induced changes in MMP and collagen production in the skin. In addition, syringaresinol contains several hydroxyl groups which are prone to forming glycosides. Several lignan glycosides have been studied as promising cosmeceuticals [27]. Overall, the present study showed that syringaresinol has UVA-protective effects in keratinocytes and dermal fibroblasts via suppression of MMP expression.

## 4. Materials and Methods 

### 4.1. Synthesis of (±)-Syringaresinol (SYR)

A pale green solution of sinapyl alcohol (0.321 g, 1.53 mmol), H_2_O (32 mL) and CuSO_4_∙5H_2_O (0.381 g, 1.00 eq) was stirred at room temperature (20 °C) in a photo-reactor. After 48 h, the reaction mixture was extracted with ethyl acetate (5 mL) twice. The combined organic layer was dried over MgSO_4_ and concentrated under reduced pressure. The residue was crystallized in EtOH to give a white solid (0.131 g, 41.0%).

^1^H NMR: (500 MHz, CDCl_3_) δ 6.57 (s, 4H), 5.53 (s, 2H), 4.72 (d, *J* = 3.0 Hz, 2H), 4.29–4.26 (m, 2H), 3.91–3.89 (m, 14H), 3.09 (s, 2H).

^13^C NMR: (125 MHz, CDCl_3_) δ 147.3, 134.5, 132.2, 102.9, 86.2, 72.0, 56.5, 54.5.

HRMS (ESI positive): [M+Na]^+^ calculated for C_22_H_26_O_8_Na 441.1526 found 441.1514

### 4.2. Cell Culture

HaCaT keratinocytes (300493; Cell Line Service, Eppelheim, Germany) were cultured in Dulbecco’s modified Eagle medium with 10% fetal bovine serum (FBS), and cells were kept in 37 °C incubators with an atmosphere containing 5% CO_2_ between the experiments. Human dermal fibroblasts (HDFs) (C-12302; PromoCell, Heidelberg, Germany) were cultured in Fibroblast Growth Medium (C-23020, PromoCell) and kept in 37 °C incubators with an atmosphere containing 5% CO2 between the experiments.

### 4.3. UVA Irradiation of HaCaT Keratinocytes and HDFs

HaCaT keratinocytes and HDFs were irradiated by UVA using a Bio-Sun UV Irradiation System (Vilber Lourmat, Marine, France) fitted with a UVA source designed for microplates. Cells grown in microplates were irradiated at a 10 J/cm^2^ UVA dose. Cells were irradiated in phosphate-buffered saline (PBS) without the plastic lid. When the irradiation received matched the desired programmed energy, the UVA irradiation stopped automatically, and, subsequently, the cells were incubated with their own growth medium without FBS until analysis.

### 4.4. Enzyme-Linked Immunosorbent Assay (ELISA)

Releases of MMP-1 and procollagen Iα1 in UVA-irradiated HaCaT keratinocytes and HDFs were analyzed by ELISA. Cells were pre-incubated in 6-well plates for 24 h and washed with PBS prior to UVA (10 J/cm^2^) exposure. Following UVA irradiation, the cells were treated with or without different concentrations (1, 5, 20 μM) of SYR for 24 h. Cell culture medium from each well were analyzed for its MMP-1 and type Iα1 procollagen contents per manufacturer’s instructions for the ELISA kit (R&D systems, Inc., Minneapolis, MN, USA).

### 4.5. Determination of MMP-1 Activation

MMP-1 activation was assessed using a quenched fluorogenic-substrate-based enzyme activity assay kit (Fluorokine E human active MMP-1 fluorescent assay kit; F1M00, R&D Systems, Minneapolis, MN, USA) according to the manufacturer’s protocol. MMP-1 activation was given as the active MMP-1 levels (ng/mL) in the cell culture medium.

### 4.6. Reverse Transcription Polymerase Chain Reaction (RT-PCR) 

Total RNA was isolated from nonirradiated and irradiated (UVA, 10 J/cm^2^) HaCaT keratinocytes and HDFs using TRIzol^®^ reagent (Invitrogen; Thermo Fisher Scientific, Inc.). RNA (2 μg) and oligo(dT) were mixed in RNase-free water for the cDNA synthesis. Synthesis was performed in a thermo cycler (T100; Bio-Rad Laboratories, Inc., Hercules, CA, USA) with an initial denaturation of the mix at 70 °C for 5 min and cooing immediately followed by the preparation of a master mix containing 1X RT buffer, 1 mM dNTPs, 500 ng oligo(dT), 140 units M-MLV reserve transcriptase and 40 units RNase inhibitor. The remaining cycles were 42 °C for 60 min and 72 °C for 5 min.

For RT-PCR analysis, the target cDNA was amplified using the sense and antisense primers as previously noted [28]. The amplification was carried out with 30 cycles; each cycle consisted of 95 °C for 45 s, 60 °C for 1 min and 72 °C for 45 s. The final PCR products were separated by agarose gel (1.5%) electrophoresis for 30 min at 100 V. Staining of gels were carried out with 1 mg/mL ethidium bromide and the images of the stained gels were taken under UV light using a Davinch-Chemi imager™ (CAS-400SM, Seoul, Korea).

### 4.7. Western Blotting

Protein levels were investigated using immunoblotting according to common Western blot protocols. HaCaT keratinocytes and HDFs were cultured in 6-well plates up to 80% confluence and exposed to UVA (10 J/cm^2^) irradiation. Next, SYR (1, 5, 20 μM) was added to the wells and incubated for 6, 12 or 24 h. Following incubation, wells were aspirated, and cells were lysed by vigorous pipetting in 1 mL of RIPA buffer (Sigma–Aldrich, St. Louis, MO, USA) at 4 °C. The nuclear fraction extraction was carried out using NE-PER^TM^ Nuclear Extraction Kit (Catalog No. #78835; Thermo Fisher Scientific, Waltham, MA, USA) according to manufacturer’s instructions. Protein content of the lysates was measured with a BCA protein assay kit (Thermo Fisher Scientific, Rockford, IL, USA) following kit’s protocol. The same amount (20 μg) of protein from each well was loaded onto 10% SDS-polyacrylamide gel and run at 100 V. Proteins on gel were then transferred onto a polyvinylidene fluoride membrane (Amersham, GE Healthcare, Little Chalfont, UK) using a wet system run at 100 V for 1 h at 4 °C. Next, membrane blocking was carried out by incubating membranes for 1 h at room temperature in 5% skimmed milk for blocking. Blocked membranes were washed with 1× TBST and incubated with primary antibodies of specific proteins (diluted 1:1000) in primary antibody dilution buffer containing 1× TBST with 5% bovine serum albumin overnight at 4˚C. Membranes were then incubated with horseradish-peroxidase-conjugated secondary antibodies (diluted 1:1000) specific to the primary antibody source organism at room temperature for 1 h. Detection of proteins on blotted membranes was achieved using a ECL Western blot detection kit (Amersham) according to the manufacturer’s instructions. Protein bands were imaged with CAS-400SM Davinch-Chemi imager™ (Davinch-K).

### 4.8. Statistical Analysis

All numerical data were given as the mean ± standard deviation of three separate experiments carried out in triplicates. Statistical differences between the means of the sample groups were calculated by the analysis of variance (ANOVA) followed by Duncan’s multiple range test using SAS v9.1 software (SAS Institute, Cary, NC, USA). Any statistically significant difference between the groups were determined at *p* < 0.05 level. 

## 5. Conclusions

In conclusion, the current study demonstrated that SYR attenuated UVA-induced changes in MMP-1 and collagen production as well as MMP-1 activation in HaCaT keratinocytes and HDFs. Results indicated that SYR inhibited MMP-1 expression by suppressing UVA-stimulated phosphorylation of p38 and JNK MAPKs and their downstream effectors c-Fos and c-Jun, which regulates the AP-1-activated MMP-1 expression. In addition, SYR was also suppressed the UVA-induced increase in inflammatory cytokine levels. Therefore, the present study suggests that SYR is a potential lead compound for cosmeceutical development against UVA-induced photoaging. Considering previous reports on beneficial and protective effects of syringaresinol on human skin, futures studies that focus on the in-vivo efficiency of SYR and its detailed action mechanisms are urged for the development of SYR-based cosmeceuticals.

## Supplementary Materials

Supplementary materials are available online at https://www.mdpi.com/1422-0067/21/11/3981/s1.
