# Supplementary material for: Syringaresinol Inhibits UVA-Induced MMP-1 Expression by Suppression of MAPK/AP-1 Signaling in HaCaT Keratinocytes and Human Dermal Fibroblasts
Source: Int J Mol Sci. 2020 Jun 1;21(11):3981. doi: 10.3390/ijms21113981 (PMC7312901; doi:10.3390/ijms21113981)
Supplement: Supplementary file 1 [file ijms-21-03981-s001.pdf]

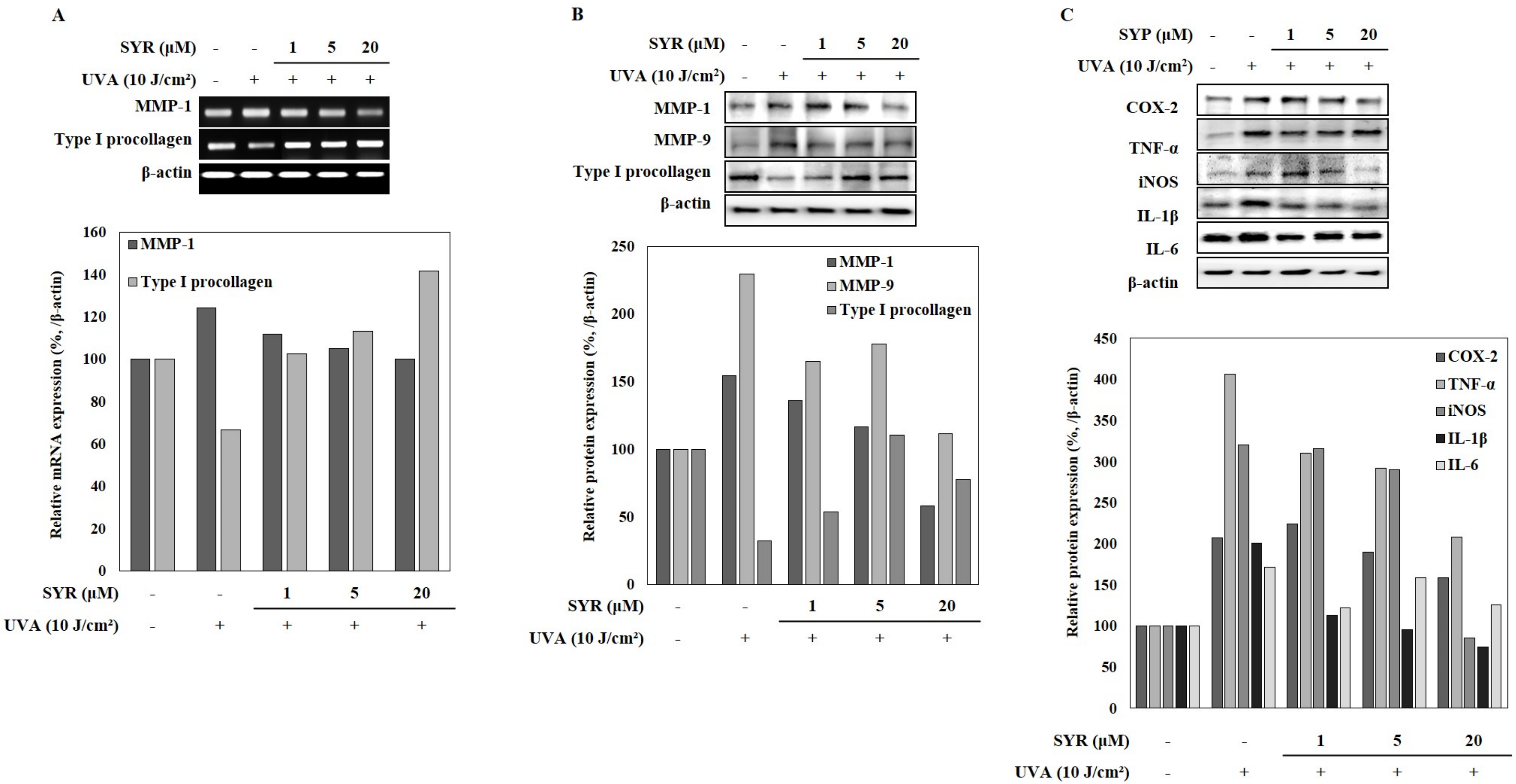

Figure S1. Quantification of Figure 2.

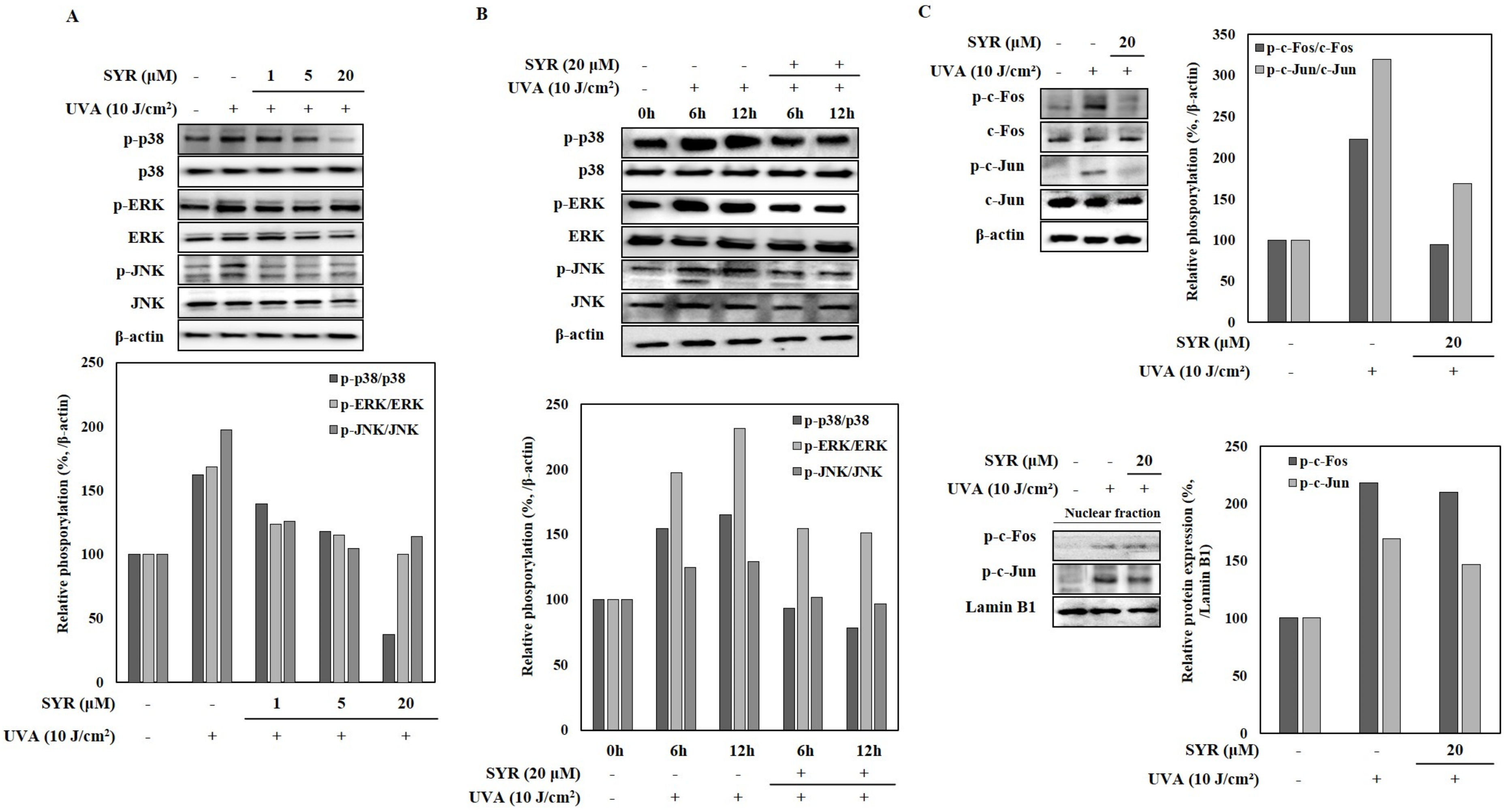

Figure S2. Quantification of Figure 3.

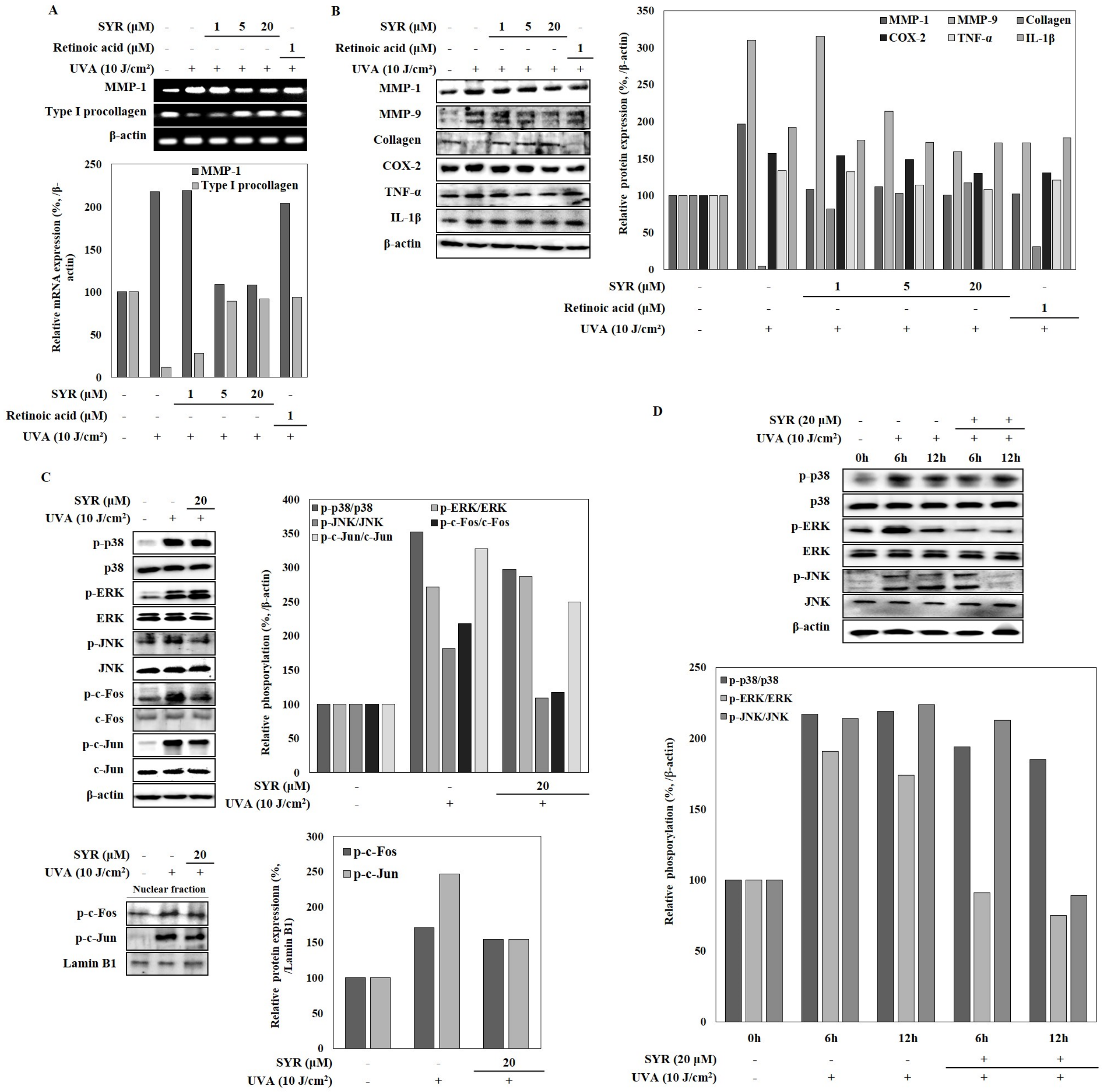

Figure S3. Quantification of Figure 5.
